# Prevalence of intestinal parasites versus knowledge, attitude and practices (KAPs) with special emphasis to *Schistosoma mansoni* among individuals who have river water contact in Addiremets town, Western Tigray, Ethiopia

**DOI:** 10.1371/journal.pone.0204259

**Published:** 2018-09-25

**Authors:** Alganesh Gebreyohanns, Melese Hailu Legese, Mistire Wolde, Gemechu Leta, Geremew Tasew

**Affiliations:** 1 Department of Medical Laboratory Sciences, College of Health Sciences, Addis Ababa University, Addis Ababa, Ethiopia; 2 Ethiopian Public Health Institute, Leishmaniasis Research Laboratory, Addis Ababa, Ethiopia; George Washington University School of Medicine and Health Sciences, UNITED STATES

## Abstract

**Background:**

Intestinal parasite infections are major public health problems in resource-limited countries that adversely affect the well-being of millions. Among these, intestinal schistosomiasis is a serious public health problem in tropical and sub-tropical countries.

**Methods:**

A Community based cross sectional study was conducted from February to April 2017 in Addiremets town, Ethiopia. Socio-demographic associated risk factors and knowledge, attitude and practices of individuals regarding intestinal parasite infection including schistosomiasis were collected from 411 study participants using pretested structured questionnaires. From each study participant, a fresh stool sample was collected and direct microscopy, formol-ether concentration and Kato- Katz techniques were performed. Snails were checked and collected from the nearby study area river. The collected data was entered and analyzed using SPSS version 20. Bi-variant and multiple logistic regressions were used for correlation analysis. A P <0.05 was considered as statistically significant.

**Result:**

The overall intestinal parasite prevalence was 51.3% (211/411). The most prevalent parasites were *S*. *mansoni* 26.3%(108/411) and Hookworm *23*.1%(95/411). The prevalence of intestinal parasites among males and females were 54.1%(131/242) and 47.3%(80/169) respectively. The highest proportion of parasite infection was reported among the age group of 5–9 year old participants, at 70.6%(36/51). The prevalence of *S*. *mansoni* was 26.3% (108/411) with mean infection intensity of 218 eggs per gram (range: 24 to 1728). Among study participants, 94.4% had good knowledge while 35.9% of them had poor practices towards intestinal parasite and Schistosomes infection prevention.

**Conclusion:**

High prevalence of intestinal parasitic infection was observed in Addiremets town and the most common parasites identified were *S*. *mansoni* and Hookworm. Most study participants had light infection intensity of Schistosomiasis, Ascariasis and Hookworm infection. Majority of the participants in the study area had good knowledge and positive attitude about intestinal parasitic infection and schistosomiasis control. Shells of *Biomphalaria* species, *Bulinus* species and *Physa* species were collected from the Mytsaeda river shore.

## Introduction

Globally, it is estimated that around 3.5 billion individuals have been infected with intestinal parasites where 450 million individuals developed the diseases [1]. The major etiologic agents of intestinal parasitosis are *Schistosoma mansoni*, *Entamoeba histolytica*, *Giardia lamblia*, *Ascaris lumbricoides*, *Trichuris trichiura*, Hookworms, *Hymenolepis species*, *Taenia species*, *Strongyloides stercoralis and Enterobius vermicularis* [2]. Among the leading causes 438.9 million people were infected with Hookworms, 819.0 million with *A*. *lumbricoides* and 464.6 million with *T*. *trichiura* in 2010. Among the 4.98 million years lived with disability (YLDs) attributable to STH, 65% were attributable to Hookworms, 22% to *A*. *lumbricoides* and the remaining 13% to *T*. *trichiura* [3, 4].

Of schistosomiasis, the most prevalent form is intestinal schistosomiasis which is a serious public health problem in tropical and subtropical parts of the world especially in areas where there is limited access to safe drinking water and poor sanitation [5, 6]. In children, schistosomiasis can cause anemia, stunting and a reduced ability to learn and in some cases may result in death [7, 8]. River water can be contaminated with human feces containing Schistosomes eggs which release miracidium upon contact with water. The released miracidium will swim and enter an intermediate host where it develops into the infective stage known as cercaria. The snail continuously sheds cercaria into water, where people will acquire the infection after coming into contact with cercaria infested water [8, 9].

These intestinal parasitic infections are highly abundant in Ethiopia where is estimated that one third of its population is infected with *A*. *lumbricoides*. It was also reported that one quarter of the population is infected with *T*. *trichiura* and the other with Hookworms [9]. Moreover, intestinal schistosomiasis which is caused by S. mansoni is among the highest public health problems in Ethiopia. It is widely distributed in some parts of the country and it is more prevalent in areas 1300–2200m above sea level (asl) [10]. The Mollusca *Biomphalaria sudanica* (*B*. *sudanica*) and *Biomphalaria pfeifferi* (*B*. *pfeifferi*) are the main intermediate hosts for *S*. *mansoni* and *B*. *pfeifferi* has a wider geographical distribution in Ethiopia [11].

Community awareness and involvement in control strategies are considered as one of the most important tools for the success and sustainability of any control program. Assessing knowledge, attitude and practice of individuals regarding Schistosomes risk factors, transmission, intermediate host (snail) in the community have a great importance for identifying, designing and implementing effective community-based interventions [12, 13].

In Ethiopia, only a few studies have been conducted on individuals who have river water contact and whose residence is around the river. Moreover, data on intestinal parasitosis especially about intestinal schistosomiasis is so limited in the town. Hence, this study aimed to determine the prevalence of intestinal parasites and knowledge, attitude and practices (KAPs) among individuals who have river water contact with special emphasis on *S*. *mansoni* in the town of Addiremets, Ethiopia.

## Materials and methods

A community based cross sectional study was conducted in Addiremets town, Western Tigray, Ethiopia, between February and April 2017. Addiremets town is located 1075 km from the capital city of Ethiopia, Addis Ababa and is located at 13^0^45^ꞌ^N latitude, 37^0^19^ꞌ^ E longitude with an elevation of 1,870 m (6,140 ft) above sea level. Based on the 2007 Census conducted by the Central Statistical Agency, Addiremets has a total population of 5,203 of which 2,446 are male and 2,757 are female [14]. All individuals whose residence was Addiremets, and who had river water contact at the study site were taken as source population. Those individuals who lived for at least six months, who had contact with the river (Mytsaeda River) and who gave consent to provide specimens were enrolled in the study. However, individuals who took medication for intestinal parasites including S. *mansoni* within the last three months and who were not willing to participate in the study were excluded.

The sample size was calculated using single population proportion formula n = Z2α/2 P (1- P)/ d2 [15] by taking a proportion value of 0.42 from a previous study conducted on schistosomiasis infection by Desta in Ethiopia [16] where “n” is the minimum number for sample size, “Z” is standard value which is Z = 1.96, “d” is marginal error at 95% confidence interval and marginal error of 5%(0.05) After the calculation, the sample size was found to be 374 and with a 10% non- respondent rate the final sample size became 411. Systematic random sampling technique was applied to select households in the town. Based on the 2007 census conducted, this town has 1,432 households and the average family size was 3.51. Based on this, 411 individuals were selected from (411/3.51) 118 households and the ‘‘K” value became 12 (1432/3.51). The first household was selected using a lottery method and every 12^th^ house number was included. If the 12^th^ house was not convenient, the next household was sampled. In each household, 4 individuals were recruited conveniently. If the number of household members was less than 4, the remaining was taken from the next household. Socio- demographic, associated risk factors and KAPs related data regarding intestinal parasitic infection including schistosomiasis were captured using a pretested structured questionnaire. Each study participant gave fresh stool samples the following day at the primary hospital called Addiremets Primary Hospital. After instructing each study participant about fresh stool specimen collection, a dry, clean, leak-proof, detergent free and labeled stool cup was delivered to bring an adequate amount of fresh stool. Immediately after collection, some portion of the stool samples were processed by direct microscopy and kato-katz technique. The remaining portion was preserved in sodium acetate acetic acid formalin (SAF) solution for concentration technique. Finally, the shells of the snail, intermediate hosts of *S*. *mansoni* were collected from the shores of the Mytsaeda River at the site of human-water contact using forceps.

### Direct microscopic examination

Direct microscopy was performed at the site of collection (primary hospital) within 30 minutes of sample receipt for possible detections of parasites such as ova of *S*. *sterocoralis*, motile trophozoite of *E*. *histolytica/dispar* and *G*. *lamblia*, parallel to helminths ova, cysts and oocysts of intestinal protozoa. A direct wet mount was prepared by emulsifying approximately 5g of stool using a drop of physiological saline on a slide.

### Formal-ether concentration technique

Approximately 4gm of feces was emulsified in formol water suspension which was strained to remove large fecal particles. Into the strained feces, ether or ethyl acetate was added and then the mixed suspension was centrifuged. After discarding the supernatant, the sediment was examined under the microscope for cysts, oocysts, eggs and larvae of intestinal parasites.

### Kato-Katz technique

For microscopic examination of intestinal helminths and parasite intensity determination, Kato- Katz technique was performed by preparing stool smears on slides. This was done by taking 41.7 grams of stool to determine the intensity of the infection in terms of eggs per gram of stool (epg) on each slide.

### Quality assurance and quality control

The questionnaire was pretested using 5% of study participants in the town to check the necessary information was written properly and could be easily understood. The amount of the stool and the absence of contamination with urine and soil were supervised by the principal investigator during sample collection. All reagents used for laboratory analysis were checked by positive and negative control samples. Standard Operating Procedures (SOPs) were strictly followed during the course of sample collection, preservation, transportation, processing and examination of Kato-kaz thick smears, formal-ether concentration technique and wet mount technique. The findings of each laboratory techniques were recorded carefully.

### Data analysis and interpretation

Data was entered, checked and analyzed using SPSS version 20 software. The descriptive statistics (mean, percentages or frequency) were calculated. The bi-variant logistic regression analysis was used to assess the relation between dependent variables and independent variables. Variables showing significant association (p-value < 0.05) were further analyzed using multiple logistic regressions. Finally, the results were presented in text, charts, graphs, pictures and tables.

### Ethical considerations

Ethical clearance was obtained from the Department of Research and Ethical Review Committee (DRERC) of Medical Laboratory Science, School of Allied Health Sciences, College of Health Science, and Addis Ababa University. A written permission letter was also obtained from the local health bureau. The aim of the study, benefits and rights were explained to study participants/ relatives and written informed consent was obtained. Stool samples were taken from those individuals who were volunteers and signed the consent form. Any personal information that was obtained during the study was kept confidential. Persons who were found to be positive for intestinal parasites including *S*. *mansoni* were treated accordingly.

## Results

### Socio-demographic findings

A total of 411 individuals with ages ranging from 5 to 70 years were included in this study. The mean age of study participants was 21.72 with a standard deviation of ±11.15. Out of this, 58.9% (242/411) were males and 41.1% (169/411) were females. 21.7% (89/411) of the study participants fall within the age category of 15–19 years followed by 19.2% (79/411) falling in the age group of 20–24 years. Regarding their occupation, students took the highest proportion 64.7% (267/411) followed by jobless mothers 12.7% (52/411) (Table 1).

**Table 1:** Socio-demographic data of the study participants of Addiremets town.

Socio-demographic characteristics		No. of study participants	Percent
**Sex**	Male	242	58.9
	Female	169	41.1
**Age group**	5–9	51	12.4
	10–14	59	14.4
	15–19	89	21.7
	20–24	79	19.2
	25–29	43	10.5
	30–34	36	8.8
	35–39	22	5.4
	40–44	12	2.9
	45–49	10	2.4
	≥50	10	2.4
**Religion**	Orthodox Christian	367	89.3
	Muslim	44	10.7
**Occupation**	Employed	25	6.1
	Student	267	64.7
	Farmer	42	10.4
	House wife	52	12.7
	Merchant	25	6.1
**Education Status**	Illiterate	70	17.0
	Primary	169	41.1
	Secondary	99	24.1
	Tertiary	22	5.4
	College	47	11.4
	University	4	1.0
**Total**		**411**	**100**

### The prevalence of intestinal parasites in Addiremets town

The overall prevalence of intestinal parasites was 51.3% (211/411). The prevalence of intestinal parasites among males and females was 54.1% (131/242) and 47.3% (80/169) respectively. The highest proportion 70.6% (n = 36/51) of intestinal parasites was detected among the age group of 5–9 year participants. Intestinal parasitic infection was significantly associated with age groups of 5–9 years, 10–14 years and 15–19 years [(AOR10.302; 95% CI 1.482–71.621, P = 0.018), (AOR 8.370; 95% CI 1.227–57.113, P = 0.030), (AOR 6.582; 95% CI 1.008–42.993, P = 0.049)] respectively. However, gender, occupation and educational status didn’t show any statistically significant associations (Table 2).

**Table 2:** The distribution and association of intestinal parasites with socio demographic data.

Socio-demographic characteristics		total	No(%) of positive for intestinal parasites	COR95%CI	p-value	AOR 95%CI	p-value
Sex	Male	242	131(54.1)	1.313(0.886–1.947)	0.175		
	female	169	80(47.3)	1	1		
Age group	5–9	51	36(70.6)	9.600(1.821–50.613)	0.008*	10.302(1.482–71.621)	0.018*
	10–14	59	39(66.1)	7.800(1.512–40.233)	0.014*	8.370(1.227–57.113)	0.030*
	15–19	89	54(60.7)	6.171(1.238–30.776)	0.026*	6.582(1.008–42.993)	0.049*
	20–24	79	41(51.9)	4.316(0.862–21.615	0.075		
	25–29	43	15(34.9)	2.143(0.403–11.401)	0.372		
	30–34	36	15(41.7)	2.857(0.530–15.410)	0.222		
	35–39	22	3(13.6)	0.632(0.088–4.532)	0.648		
	40–44	12	2(16.7)	0.800(0.091–7.002)	0.840		
	45–49	10	4(40)	2.667(0.361–19.712)	0.337		
	>50	10	2(20)	1	1		
Occupation	Employed	25	7(28)	1.231(0.347–4.371)	0.747		
	Student	267	163(61)	4.963(1.919–12.836)	0.001*	1.421(0.392–5.157)	0.593
	Farmer	42	16(38.1)	1.705(0.554–5.250)	0.352		
	Housewife	52	19(36.5)	1.880(0.639–5.532)	0.252		
	Merchant	25	6(24)	1	1		
Educational status	Illiterate	70	24(34.3)	0.522(0.069–3.938)	0.528		
	Primary	169	98(58)	1.380(0.190–10.033)	0.750		
	Secondary	99	51(51.5)	1.062(0.144–7.845)	0.953		
	Tertiary	22	11(50)	1.000(0.119–8.421)	1.000		
	Higher level	51	27(53)	1	1		
Total		411	211				

(COR: crud odds ratio; CI: confidence interval; AOR: adjusted odds ratio, *; Statistical significant association)

**Fig 1:**
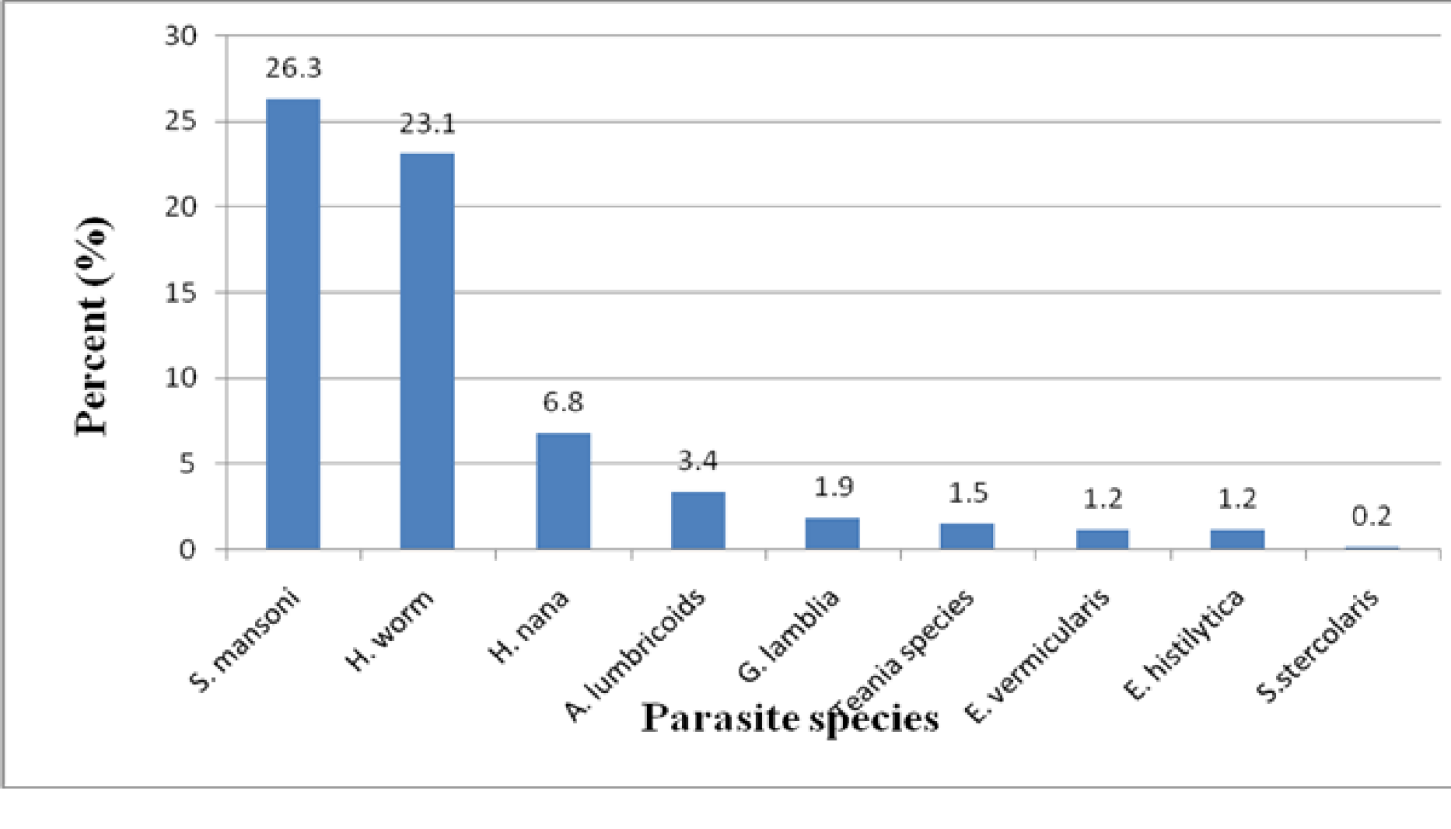
Species distribution of intestinal parasites detected, Addiremets town, 2017.

**Fig 2:**
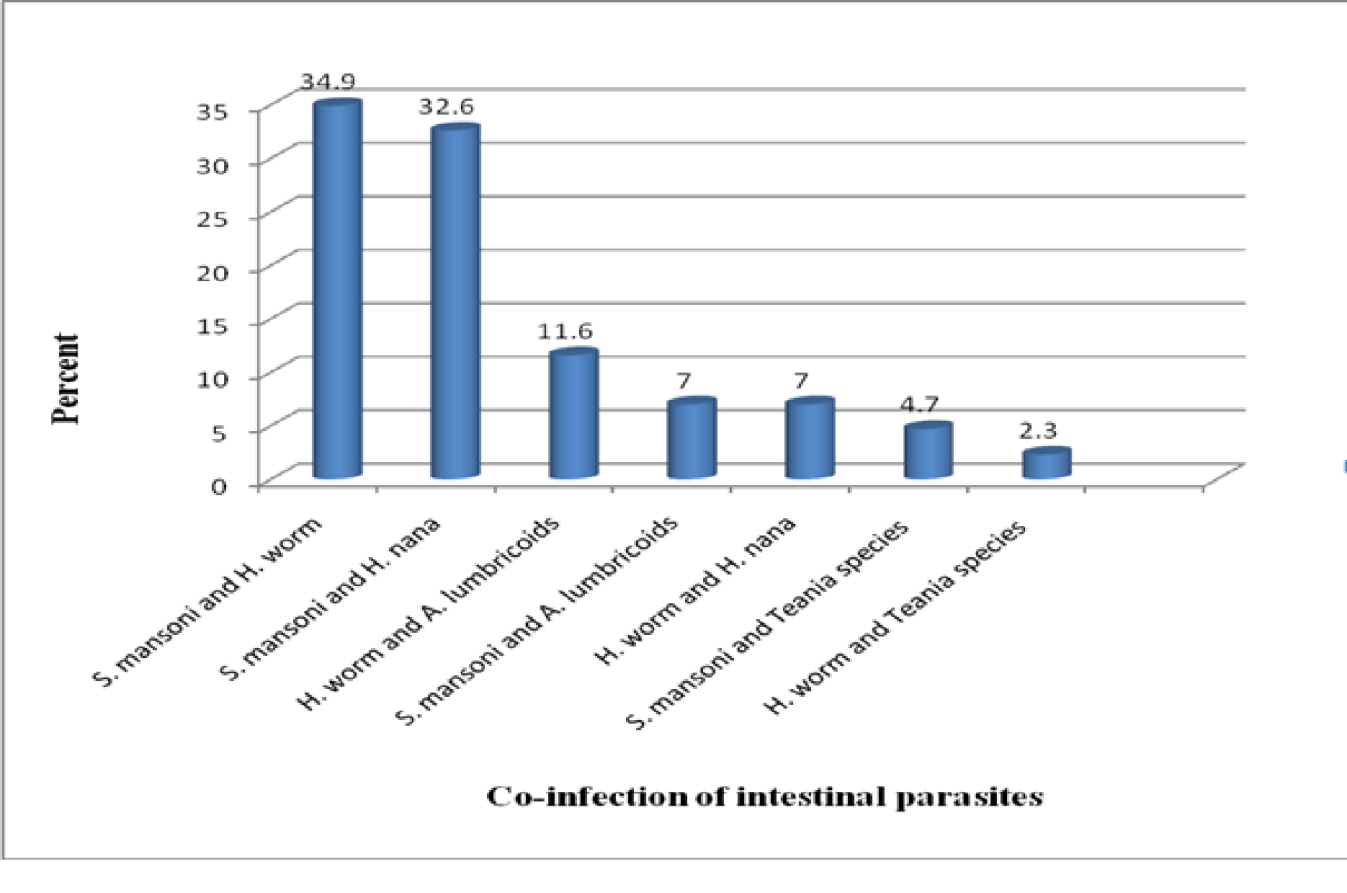
Co-infection of intestinal parasites, Addiremets town, 2017.

In this study, nine intestinal parasite species were identified and of them *S*. *mansoni* 26.3% (108/411) was the most common (“Fig 1”). The overall prevalence of Soil-transmitted helminths was 26.5% (109/411) in which Hookworms and *A*. *lumbricoides* accounted for 23.1% (95/411) and 3.4% (14/411) respectively. In our study, a total of 20.4% (43/211) co-infections were found where *S*. *mansoni* and Hookworms co-infection was 34.9% (15/43) followed by *S*. *mansoni* and *H*. *nana* co-infection 32.6% (14/43) (“Fig 2”).

### Infection intensity of *S*. *mansoni*, *Hookworms* and *A*. *lumbricoides*

The mean egg per gram (epg) of *S*. *mansoni*, Hookworms and *A*. *lumbricoids* were 218 epg (Range: 24 to 1728), 563 epg (Range: 24 to 3360) and 5292 epg (Range: 672 to 14,400), respectively. For *S*. *mansoni* of the 108 individuals who were positive, the egg count was performed for 72 study subjects because the remaining study subjects were positive either in direct microscopy or in formal-ether concentration technique or in both but not in Kato-Katz technique. Therefore, of the 72 individuals 35(48.6%) had light infection, 25 (34.7%) had moderate infection and 12(16.7%) had heavy infection. For Hookworms, of 95 positive samples 87 of them were detected using the Kato-Katz technique. Seventy-seven (88.5%) had light infection, 9(10.3%) had moderate infection) and 1(1.2%) had heavy infection (Table 3).

**Table 3:** Infection intensity of *S*. *mansoni*, *H*. *worm* and *A*. *lumbricoides*.

Socio-demographic		Infection intensity								
		*S*. *mansoni n (%)*			*Hookworm n (%)*			*A*. *lumbricoids n (%)*		
		Light(1–99)	Moderate (100–399)	Heavy (>399)	Light(1–1999)	Moderate(2000–3999)	Heavy (>3999)	Light(1–4999)	Moderate(5000–49,999)	Heavy(>49,999)
**Sex**	Male	13(50.0)	10(38.5)	3(11.5)	22(28.6)	3(33)	1(100)	4(50)	2(50)	0
	Female	22(47.8)	15(32.6)	9(19.6)	55(71.4)	6(66.7)	0	4(50%)	2(50)	0
**Age group**	5–9	8(22.9)	7(28.0)	4(33.3)	3(3.9)	0	0	0	2(50)	0
	10–14	8(22.9)	9(36.0)	6(50.0)	2(2.6)	1(11.1)	0	0	1(25)	0
	15–19	11(31.4)	3(12.0)	2(16.7)	28(36.4)	3(33.3)	0	3(37.0)	1(25)	0
	20–24	6(17.1)	3(12.0)	0	23(29.9)	3(33.3)	0	1(12.5)	0	0
	25–29	1(2.9)	0	0	11(14.3)	0	0	0	0	0
	30–34	0	1(4.0)	0	8(10.4)	1(11.1)	0	1(12.5)	0	0
	35–39	0	0	0	1(1.3)	1(11.1)	0	1(12.5)	0	0
	40–44	1(2.9)	0	0	0	0	0	0	0	0
	45–49	0	1(4.0)	0	1(1.3)	0	1(100)	2(25.0)	0	0
	≥50	0	1(4.0	0	0	0	0	0	0	0

### Prevalence of *S*. *mansoni* in Addiremets town

In our study, *S*. *mansoni* prevalence was 26.3% (108/411). Among 242 male and 169 female participants 27.7% (67/242) and 24.3% (41/169) were positive for *S*. *mansoni* respectively. The distribution of *S*. *mansoni* infection with age groups showed that the age group of 10–14 years old (61%) and 5–9 year olds (54.9%) were affected with *S*. *mansoni* infection. With regard to occupation students were more affected with *S*. *mansoni* with infection rate of 36.7%(98/267) (Table 4).

**Table 4:** Distribution of *S*. *mansoni* infection by socio demographic data.

Socio-demographic characteristics		Group total	No. of positive cases for *S*. *mansoni*	Percent
**Sex**	Male	242	67	27.7
	Female	169	41	24.3
**Age group**	5–9	51	28	54.9
	10–14	59	36	61
	15–19	89	22	24.7
	20–24	79	14	17.7
	25–29	43	1	2.3
	30–34	36	3	8.3
	35–39	22	0	0
	40–44	12	2	16.7
	45–49	10	1	10
	≥50	10	1	10
**Occupation**	Employed	25	1	4
	Student	267	98	36.7
	Farmer	42	4	9.5
	House wife	52	3	5.8
	Merchant	25	2	8
**Education Status**	Illiterate	70	4	5.7
	Primary	169	73	43.2
	Secondary	99	21	21.2
	Tertiary	22	2	9.1
	**Higher level**	**51**	**8**	**15.7**

Individuals who wore open shoes had a higher rate of infection than who wore closed shoes (AOR 8.126; 95% CI 1.789–36.904, P = 0.007). Similarly, individuals who swim three or more times a week had a higher rate of infection than who swim once a week (AOR 2.506; 95% CI 1.096–5.730, P = 0.029). (Table 5)

**Table 5:** Association of *S*. *mansoni* infection with risk factors and demographic factors.

Risk factors		*S*. *mansoni*		COR (95.0% C.I.)	P-value	AOR (95.0% C.I.)	P-value	
		Negative	Positive					
**Sex**	Male	175	67	0.837(0.533–1.313)	0.438			
	Female	128	41	1	1			
**Age group**	5–9	23	28	10.957(1.291–92.970)	0.028*	4.434(0.260–75.535)	0.303	
	10–14	23	36	14.087(1.672–118.682)	0.015*	5.640(0.332–95.860)	0.231	
	15–19	67	22	2.955(.354–24.654)	0.317			
	20–24	65	14	1.938(.227–16.56	0.545			
	25–29	42	1	0.214(0.012–3.756)	0.292			
	30–34	33	3	0.818(0.076–8.842)	0.869			
	35–39	22	0	0.000(.000)	0.998			
	40–44	10	2	1.800(.139–23.374)	0.653			
	45–49	9	1	1.000(.054–18.574)	1.000			
	≥50	9	1	1	1			
**Occupation**	Employed	24	1	0.479(.041–5.652)	0.559			
	Student	169	98	6.669(1.539–28.893)	0.011*	1.478(0.161–13.584)	0.730	
	Farmer	38	4	0.932(0.145–6.010)	0.941			
	House wife	49	3	0.719(0.112–4.603)	0.727			
	Merchant	23	2	1	1			
**Swimming habit**	yes	115	76	3.883(2.418–6.235)	0.00*	1.407(0.753–2.626)	0.284	
	no	188	32					
**Place of Bathing**	Mytsaeda river	251	105	7.251(2.215–23.736)	0.01*	1.439(0.230–8.998)	0.697	
	home	52	3	1	1			
**Type of shoe**	open	224	104	8.967(3.196–25.154)	0.001*	8.126(1.789–36.904)	0.007*	
	closed	78	4	1	1			
**Washing clothes in the river**	yes	263	76	0.361(0.213–0.614)	0.00			
	no	40	32	1	1			
**Frequency of swimming per week**	Three times	44	44	3.250(1.504–7.021)	0.003*	2.506(1.096–5.730)	0.029*	
	Two times	32	20	2.031(0.864–4.775)	0.104			
	One time	39	12	1	1			
**Duration of stay during swimming**	1 hour	33	36	2.347(1.253–4.396)	0.008*	2.001(1.012–3.956)	0.046*	
	2 hour	11	7	1.369(0.487–3.849)	0.551			
	½ hour	71	33	1	1			

(**Note**: COR: crude odd ratio; CI: confidence interval; AOR: adjusted odd ratio; *; Statistical significant association)

### Knowledge, attitude and practices of study participants towards intestinal parasitosis and intestinal schistosomiasis

#### Knowledge about intestinal parasitosis and intestinal schistosomiasis

In total, 73.2% (301/411) of the study participants (older than 15 years) were interviewed for knowledge, attitude and practices (KAPs) towards intestinal parasitosis and intestinal schistosomiasis. Of these 301, 94% (283/301) had heard about intestinal parasites and 45.2% had knowledge about intestinal parasitosis prevention. (Table 6)

**Table 6:** Knowledge about intestinal parasitosis and schistosomiasis symptoms, transmission and prevention.

Knowledge variables n = 301	Yes n (%)	No n (%)	Total
Have you ever heard about intestinal parasites?	283(94)	18(6)	301
How did you hear about intestinal parasites?			
a. Radio	11(3.9)		
b. Health facility	123(43.4)		
c. Friends	82(29)		
d. School	67(23.7)		
Have you ever heard of schistosomiasis?	292(97)	9(3)	301
How is Schistosoma transmitted?			
a. Swimming in infested river water	132(43.9)	169(56.1)	301
b. Drinking dirty water	171(56.8)	130(43.2)	301
c. Playing in infested river water	43(14.3)	258(85.7)	301
d. Snail	18(6)	283(94)	301
e. By flies	0	301(100)	301
f. Eating contaminated raw food	35(11.6)	266(88.4)	301
g. Don’t know	36(12)	265(88.0)	301
This is a question for those who answered snail for the transmission. Where do snails reside?			
a. In river	11(64.7)		
b. In soil	4(23.5)		
c. Don’t know	2(11.8)		
What are the main signs and symptoms of intestinal schistosomiasis?			
a. Fever	61(20.3)	240(79.7)	301
b. Headache	8(2.66)	293(97.3)	301
c. Weakness	81(26.9)	220(73.1)	301
d. Dry cough	1(0.3)	300(99.7)	301
e. Abdominal pain / discomfort	155(51.5)	146(48.5)	301
f. Diarrhea	102(33.9)	199(66.1)	301
g. Blood in stool	79(26.2)	222(73.8)	301
h. Don’t know	87(28.9)	214(71.1)	301
Is Schistosomiasis treatable?	293(97.3)	8(2.7)	301
Where do you prefer to seek treatment for Schistosomiasis?			
a. Traditional healer	1(0.3)		
b. Health facility	298(99)		
c. Pharmacy (drug shop)	2(0.7)		
Is Schistosomiasis a preventable disease?	283(94)	18(6)	301
How do you prevent schistosomiasis?			
a. Treatment with specific medicines	54(19.1)	229(80.9)	283
b. Avoid bathing or swimming in stagnant water	128(45.2)	155(54.8)	283
c. Use of toilets	64(22.6)	219(77.4)	283
d. Provision of safe tap water	198(70)	85(30)	283
e. Avoid defecating in lakes	13(4.6)	270(95.4)	283
f. Personal hygiene	13(4.6)	270(95.4)	283
g. Don’t know	1(0.4)	282(99.6)	283

96% (289/301) of study subjects understood schistosomiasis as a serious disease. However, 31.9% (96/301) of the respondents believed that playing on the soil cannot be a cause for intestinal parasite infection. The majority of respondents 85.7% (258/301) believed that swimming/bathing in river can cause Schistosomiasis (Table 7).

**Table 7:** Attitude of study participants towards intestinal parasites and intestinal schistosomiasis.

Attitude variable (n = 301)	Yes n (%)	No n (%)	Total
Do you think intestinal parasitosis is a serious disease?	283(94.0)	18(6.0)	301
Do you think to take a medication against intestinal parasitosis is important?	300(99.7)	1(0.3)	301
Do you think going to a health facility is important when you feel abdominal discomfort?	301(100)	0	301
Do you think taking traditional medication is effective to treat intestinal parasitosis?	27(9)	274(91)	301
Do you think playing in soil can cause intestinal parasitosis?	205(68.1)	96(31.9)	301
Do you think eating raw vegetables can cause intestinal parasitosis?	285(94.7)	16(5.3)	301
Do you think Schistosomiasis is a serious disease?	289(96)	12(4)	301
Do you think to take medication against Schistosomiasis is important?	300(99.7)	1(0.3)	301
Do you think swimming/bathing in river water can cause Schistosomiasis?	258(85.7)	43(14.3)	301
Do you think Schistosomiasis is treatable?	296(98.3)	5(1.7)	301

Of the study participants who responded to practices related questions, 88.0% (265/301) of the respondents had practices of swimming or bathing in river and 56.5% (170/301) defecate around the river. And, 92.7% (279/301) of respondents wash their clothes in the river (Table 8).

**Table 8:** Practice of the study participants towards intestinal parasites and intestinal schistosomiasis.

Practice variable (n = 301)	Yes n (%)	No n (%)	Total
Do you eat raw meat/vegetables?	230(76.4)	71(23.6)	301
Do you wash your hands before meal?	301(100)	0	301
Do you go to a health facility when you feel abdominal discomfort?	284(94.4)	17(5.6)	301
Do you take medications for intestinal parasitosis?	299(99.3)	2(0.7)	301
Do you wash your clothes in the river?	279(92.7)	22(7.3)	301
Do you swim/bathing in the river?	265(88.0)	36(12.0)	301
Do you defecate around the river?	170(56.5)	131(43.5)	301
Do you immediately dry your body after swimming?	9(3.0)	292(97.0)	301
Do you fetch river water for cooking/drinking?	29(9.6)	272(90.4)	301
Do you participate in mass drug treatment for intestinal parasitosis?	74(24.6)	227(75.4)	301

### Malacological survey

**Fig 3:**
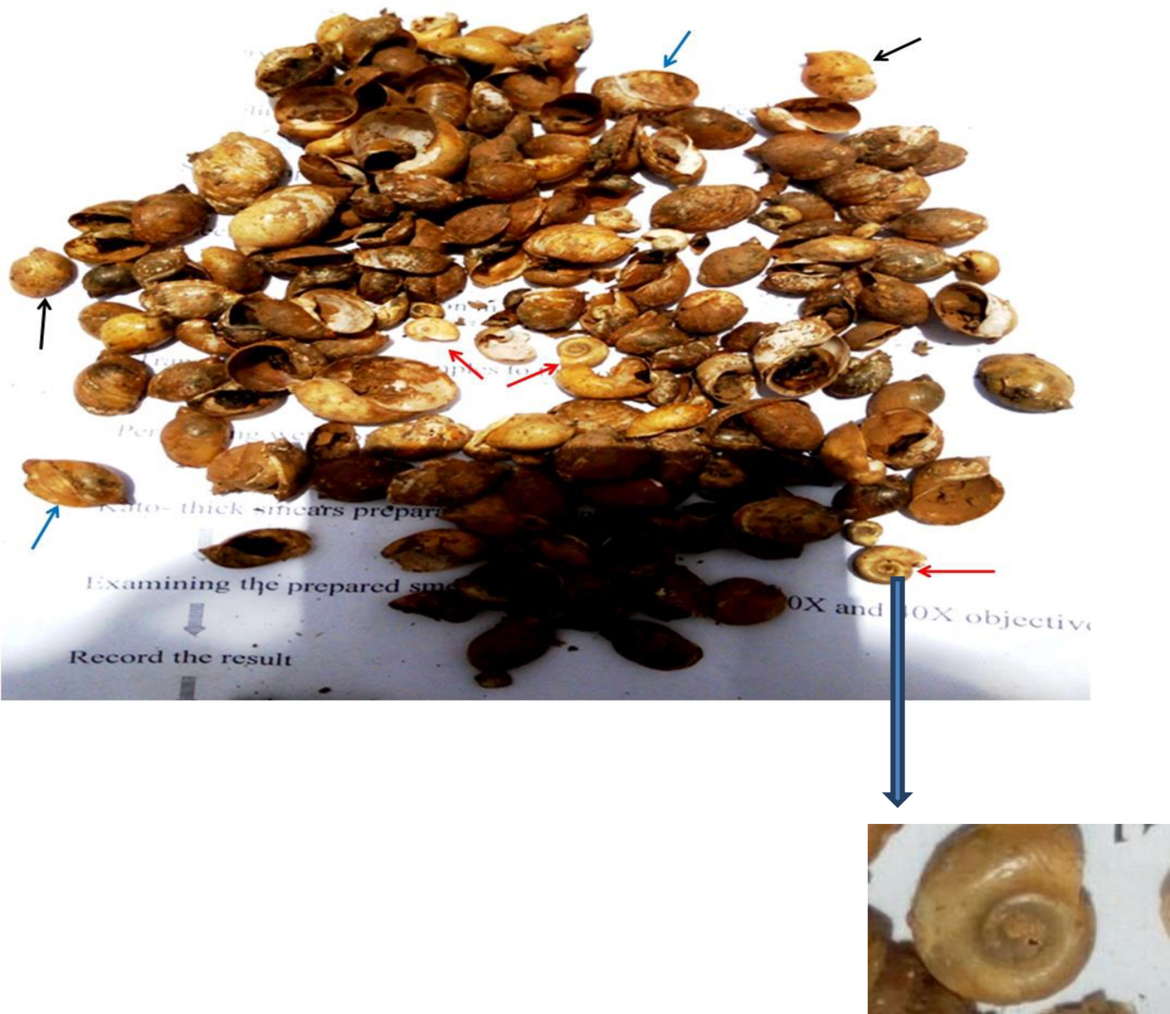
Snails collected from Mytsaeda river, Addiremets town.

Snails were surveyed in Mytsaeda River water and were collected by handpicking with forceps and gloves. Morphologically, *Biomphalaria* types of snail shells were observed among different types of snail shells collected from the river. However, no live snail was found from the site during the study period, only the shell. Identified snails were *Biomphalaria*, *Bulinus* and *Physa* species. (“Fig 3”).

**Fig 4:**
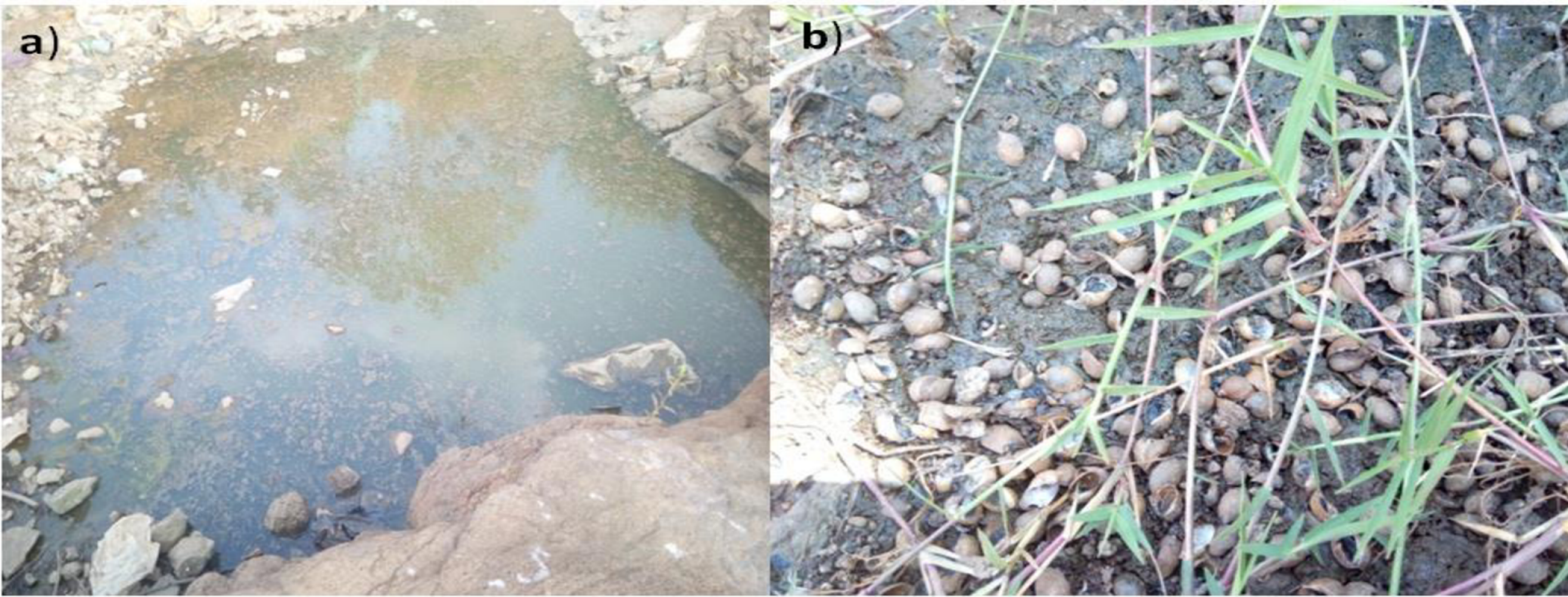
Snail habitat of Mytsaeda river, Addiremets town.

**Fig 5:**
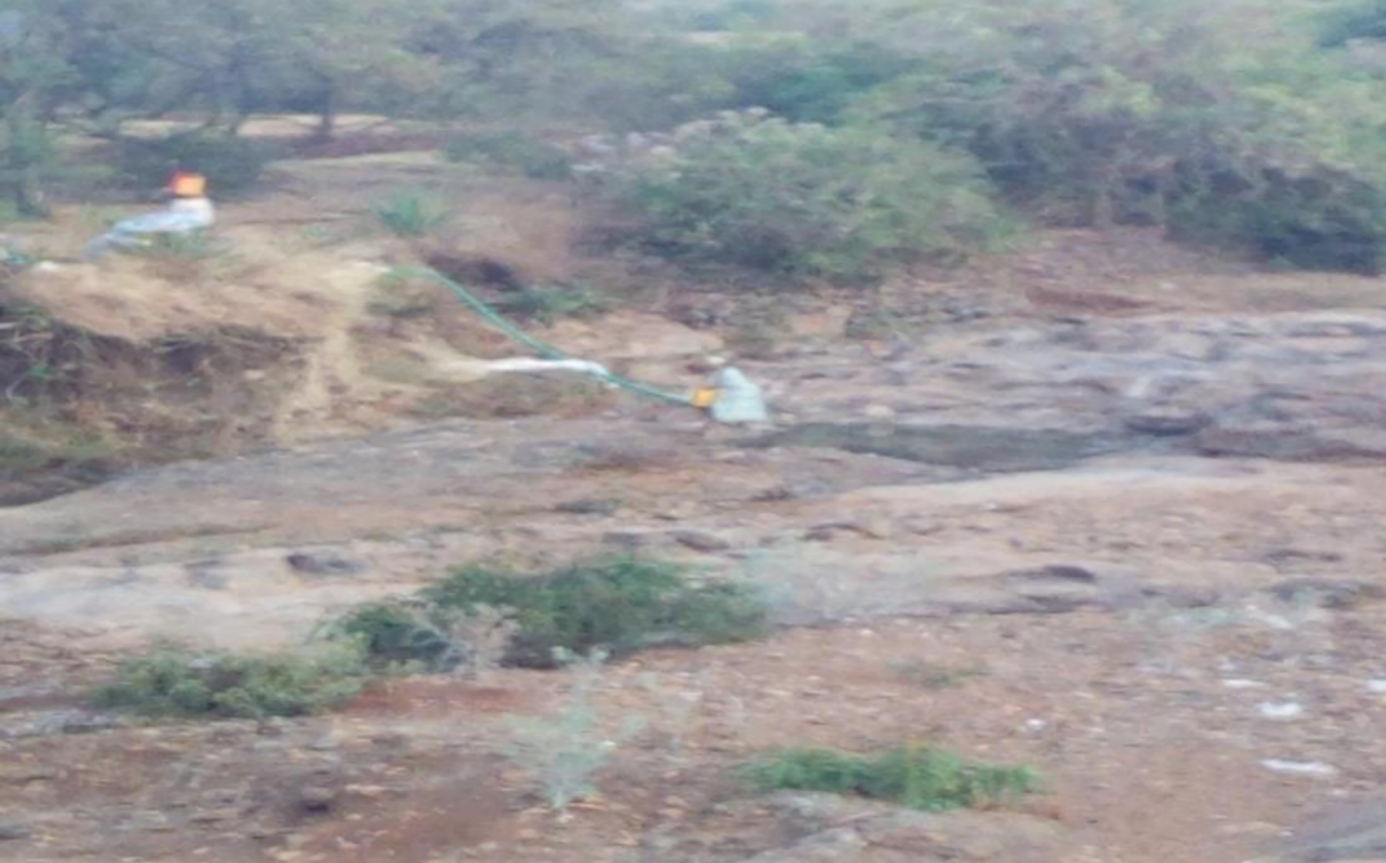
Activities of the residents (fetching water, taking water by pipe for irrigation), Addiremets town.

Observations made on physical characteristics of the stream during snail survey showed that the water was highly turbid. In the summer season it has a high volume and high speed but subsequently decreases its volume and speed, and forms a stagnant pool. During the study period it was a stagnant pool (see “Fig 4A”). In the surrounding dry areas there was grass and snail shells were found (see “Fig 4B”). Even though it was a stagnant pool, people in the surrounding area were using the river for irrigation and to wash their cloths as well as fetch water for other purposes (See “Fig 5”).

## Discussion

Assessing the prevalence of intestinal schistosomiasis and knowledge, attitude and practices (KAPs) of individuals is important for intervention and control programs. Hence, the present study was aimed to assess the prevalence of intestinal parasites and knowledge,attitude and practices (KAPs) of individuals towards intestinal parasites especially *S*. *mansoni* inAddiremets town.

The high prevalence (51.3%) of intestinal parasites in Addiremets town, Western Tigray was comparable with previous studies from Ethiopia [17, 18] however it was higher than reports from Nigeria and other parts of Ethiopia, where the reported prevalence was 26.2% and 28.0% respectively [19, 20] and lower compared to previous reports in other parts of Ethiopia [2, 15]. In Tigray, SW Ethiopia and Jimma, infection rates of 60.7%, 76.7% and 68.7% respectively were reported [2, 15, 21]. The difference might be due to differences in the age of the study participants, sample size, geography, study subjects, socioeconomic and hygienic conditions of the population.

In the present study, the most prevalent intestinal parasite was *S*. *mansoni* 26.3% (108/411) which was in agreement with the findings of a previous study conducted in Ethiopia from Abaye Deneba area [17]. In contrast, this result differs from other study findings conducted in Addis Ababa and Adigrat town where *A*. *lumbricoides* was the leading intestinal parasite [22, 17]. This difference may be due to geographical variation and water contact activities of the study participants in the present study.

Though the prevalence of intestinal parasites in males 131(54.1%) was apparently higher than females 80 (47.3%), the observed difference was not statistically significant (P>0.05). This was similar with the finding of a previous study conducted in Addis Ababa [22]. To the contrary, this finding was disagreed with previous studies conducted in Adigrat town, Ethiopia where the prevalence of intestinal parasitic infection was higher among females than males [17]. This difference might be due to the fact that in our study males mostly participated in outdoor activities like irrigation, farming and high frequency of swimming/bathing in river.

The highest proportion of parasites was reported among the age group of 5–9 year-old subjects with prevalence rate of 70.6%. This indicated that younger children were more exposed than other age groups since they usually play on soil, swim/bathe in river water and eat food without washing their hands. This finding is similar to a previous study’s findings conducted in Addis Ababa [22] where, the higher prevalence of intestinal parasitic infection was seen in the age groups of 0–9 year olds. However, another study from Tigray (Wukro town) showed that the prevalence of intestinal parasites in the age group of 15-18years old [2] and from East Gojam zone in the age group of 10–14 years old [1] were found to be higher. This difference might be due to variations in family supervision, environmental conditions and personal hygiene.

In this study, the intensities of Hookworm and *A*. *lumbricoides* infections were predominantly light. For Hookworm 77 (88.5%) had light infection (1–1999), 9(10.3%) had moderate infection (2000–3999) and 1(1.2%) had heavy infection (>3999). This result showed disagreement with a study done in Kenya in which the intensity of the infection was light and there were no moderate or heavy infections [23]. The difference might be due to the difference in study subjects; the participants of the study in Kenya were preschool age children. For *A*. *lumbricoides*, 8(66.7%) had light infection (1–4999) and 4(33.3) had moderate infection (5000–49,999) and no heavy infection. This result differed from a study done in Kenya in which the intensity of infection was predominantly heavy [23]. This difference could be due to difference in study subjects, microclimate and soil type.

The overall prevalence of *S*. *mansoni* in the present study was 26.3% which showed similarity with the finding of a previous study conducted in Jimma (Ethiopia) in which the prevalence rate was 26.3% [21]. Our result was higher than the prevalence rates reported from Nigeria: Kano State (8.9%) [24] and Sokoto (2.9%) [25] and from Ethiopia: North Gondar (15.9%) [26] and South Eastern Ethiopia (12.6%) [20]. This difference might be due to difference in water contact activities of the study participants and snail distribution. The result of the present study was lower than the previous studies conducted in Northwestern Tanzania, Finchaa Sugar Estate and Merebmieti which showed an infection rate of 64.3%, 37.5% and 42.2% respectively [10, 16,27]. The observed prevalence variation with previous studies from other localities might be due to the difference in water contact, behavior of the study participants, environmental sanitation, distribution of snails, local endemicity and sample size.

Moreover, the finding of the current study showed that the prevalence of *S*. *mansoni* was slightly higher among male participants than that of female participants. However, there was no statistically significant difference (P>0.05) in prevalence of *S*. *mansoni* infections and gender. This result agreed with studies conducted from Nigeria and Ethiopia [25, 21, 16, 6]. The higher infection rate in males can be explained by the fact that males mostly participated in outdoor activities and stay outdoors most of the time, swim most frequently and take baths in cercaria-infected water bodies and are more likely to acquiring infection. In the present study, the mean egg per gram (epg) of *S*. *mansoni* was 218 Epg. It was higher than the study conducted in Merebmeiti where the mean egg per gram was 86.7. This difference might be due to difference in treatment seeking behavior and countriess’ control program status. [16].

In the current study, 94.4% of study participants had good knowledge about the transmission, signs and symptoms, and prevention of intestinal parasite infection including intestinal schistosomiasis while the remaining 5.6% had poor knowledge. It was found that a majority of 292 (97%) of the respondents had heard about schistosomiasis (locally known as Bilharzia). Moreover, the results further showed that 58.1% (175/301) of the respondents were able to mention at least one mode of transmission. This result differs from the result of a study conducted in Yemen in which 92.4% of the respondents had heard about schistosomiasis and 49.8% of the respondents were able to mention at least one mode of transmission [12]. This difference may be due to socio demographic variation of the study participants.

In this study, 96% of the study participants believed that Schistosomiasis is a serious disease. But in another study conducted in Yemen 77.1% of the respondents believed that Schistosomiasis is a serious disease. The difference might be due to difference in the population segment of the study participants in which the study from Yemen was conducted in school children [12] while our study was conducted among the general population.

In the current study, 92.2% of the respondents had a practice of washing their clothes in the river. Moreover, 88% had a practice of swimming/bathing in the river. A lower finding was reported from a study conducted in Yemen in which 74.3% of the study participants had a practice of washing clothes in the river and 58.8% had a practice of swimming/bathing in the river [12]. This difference might be due to difference in availability of pipe water. In the site of current study there was no pipe water at an individual's household level.

### Limitation of the study

➢ Modified Ziehl-Neelsen (Zn) technique was not done for cryptosporidium.

## Conclusion

High prevalence of intestinal parasitic infection was observed in Addiremets town. The most common parasites in the town were *S*. *mansoni* and Hookworm. The majority of the study participants had light infection intensity of Schistosomiasis, Ascariasis and Hookworm infection. The majority of the participants in the study area had good knowledge and positive attitude about intestinal parasite infection and schistosomiasis control. In general, the majority of the participants had some good practices but they had also bad practices like bathing/ swimming and washing clothes in the contaminated river water and defecating around the river. Since shells of *Biomphalaria* species, *Bulinus* species and *Physa* species were collected from the river shore, we recommended that there would be a need for treating rivers with available molluscicides, creating awareness in the community towards latrine construction to reduce open field defecation and periodic deworming in the community.

## Supporting information

S1 File “English version questionnaire” consists of data collection tool in English language. (PDF)

S2 File “Amharic version questionnaire” consists of data collection tool in local language, Amharic. (PDF)
