# Supplementary material for: Prevalence of intestinal parasites versus knowledge, attitude and practices (KAPs) with special emphasis to Schistosoma mansoni among individuals who have river water contact in Addiremets town, Western Tigray, Ethiopia
Source: PLoS One. 2018 Sep 25;13(9):e0204259. doi: 10.1371/journal.pone.0204259 (PMC6155513; doi:10.1371/journal.pone.0204259)
Supplement: S1 File — (PDF) [file pone.0204259.s001.pdf]

Questioner (English and Amharic version)

ADDIS ABABAUNIVERSITY COLLEGE OF HEALTH SCIENCE SCHOOL OF ALLIED HEALTH SCIENCE DEPARTEMENT OF MEDICAL LABORATORY SCIENCE

Purpose of research: To determine the prevalence of Intestinal parasitosis and Knowledge, Attitude, practice in individuals who have river water contact with special emphasis on *S. mansoni* Addiremets, North-western Ethiopia.

Part one: socio-demographic characteristic

I. Questioner number_______

II. Sex: 1. Male 2. Female

III. Age in years: _______

IV. Religion: 1. Orthodox 2. Muslim 3. Protestant 4. Others specify_______

V. Occupation: 1. Employed 2. Unemployed 3. Student 4. Farmer 5. House wife 6. Merchant 7. Others Specify_________

VI. Education Status: 1. Illiterate 2. Primary (grade1-8) 3. Secondary (grade9-10) 4. Tertiary (grade11-12) 5. Higher level 6. Read and write only

VII. Duration of stay in Addiremets _________

VIII. Residence before coming to Addiremets _____________

Part two: Water contact Activities

1. Do you have latrine? a. Yes b. No
2. If yes to No 1, do the whole families use it?

   a. Yes b. No
3. If no to No 1 question, where do you defecate and dispose the fasces?

   a. Near the river b. Away from the river

   c. Using pits and dispose to the river
4. Do you bring water for cooking and drinking from Mytsaeda River?

   a. Yes b. No
5. If yes to question No 4, how many times a day do you fetch water for household? ---------
6. Do you swim in Mytsaeda River? a. Yes b. No

7. How many times per week do you swim in the river?

a. Three times a week b. two times a week c. one times a week

8. How long (period of time) you swim the river? ....hours
9. In what time do you swim?

a. 1:30-3:30 b. 3:30-5:30 c. 5:30-7:30 d. 7:30-9:30 e. 9:30-11:30

10 Do you dry your skin immediately after swimming by towel?

a. yes b. No

11 Do you have contact to Mytsaeda River while you are crossing it?

a. Yes b. No

12 Do you wash cloths in Mytsaeda River? a. Yes b. No

13 Where do you bath? a. Mytsaeda River b. home c. public bathroom

14 If the answer to question 13 is "b" from where do you obtain the water?

a. Mytsaeda River b. Pipe

c. Spring protected d. Well protected

15 Do you wear shoes? a. Yes b. No

16 If the answer to question 15 is "b" what type of shoe do you wear?

a. Open shoe b. closed shoe c. buts

Part three: Knowledge, Attitude, Practice

A. Questions designed to assess respondents' knowledge about intestinal parasitosis and intestinal schistosomiasis.

1. Have you ever heard about intestinal parasites? a. Yes b. No
2. How did you know about intestinal parasites?

a. Radio c. Friends

b. Health facility d. others (specify)

3. If yes to question No1, can you mention some of the intestinal parasites?

____

4. Have you ever heard of schistosomiasis? a. yes b. no

5. How is Schistosoma transmitted?

a. Swimming in infested river water b. drinking dirty water

c. Playing in infested river water d. snail e. by flies

f. eating contaminated raw food g. Don’t know

6. This is a question for those who answered “d” for Q.5 where does snail reside?

a. River b. Soil c. Do not know

7. What are the main signs and symptoms of intestinal schistosomiasis? (More than one response is Possible)

a. Fever b. Headache

c. Weakness d. Dry cough

e. Abdominal pain / discomfort f. Diarrhea

g. Blood in stool g. don’t know h. Others (specify)

8. Is Schistosomiasis treatable? a. Yes b. No

9. Where do you prefer to seek treatment for Schistosomiasis?

a. Traditional healer b. Health facility

c. Pharmacy (drug shop) d. Others (specify)

10. Is Schistosomiasis a preventable disease? a. Yes b. No

11. If yes to Q. 10, how do you prevent schistosomiasis?

a. Treatment with specific medicines b. Avoid bathing or swimming in stagnant water

c. Use of toilets d. Provision of safe tap water

e. Avoid defecating in lakes f. others (specify)

g. Personal hygiene h. Don’t know

B. Questions designed to assess respondents' attitude towards intestinal parasitosis and intestinal schistosomiasis.

1. Do you think intestinal parasitosis is a serious disease? a. Yes b. No
2. Do you think to take a medication against intestinal parasitosis is important?
   a. Yes b. No
3. Do you think going to health facility is important when you feel abdominal discomfort?
   a. Yes b. No
4. Do you think taking traditional medication is good to treat intestinal parasitosis?
   a. Yes b. No
5. Do you think playing in soil can cause intestinal parasitosis?
   a. Yes b. No
6. Do you think eating raw vegetables can cause intestinal parasitosis?
   a. Yes b. No
7. Do you think Schistosomiasis is a serious disease?
   a. Yes b. No
8. Do you think to take medication against Schistosomiasis is important?
   a. Yes b. No
9. Do you think swimming/bathing in river water can cause Schistosomiasis?
   a. Yes b. No
10. Do you think Schistosomiasis is treatable?
    a. Yes b. No

C. Questions designed to assess respondents, practice towards intestinal parasites and intestinal schistosomiasis

1. Do you eat raw meat/vegetable? a. yes b. No
2. Do you wash your hand before meal? a. yes b. No

3. Do you go to health facility when you feel abdominal discomfort?

a. yes b. No

4. Do you take medications for intestinal parasitosis? a. yes b. No
5. Do you wash your cloths in the river? a. yes b. No
6. Do you swim/bathing in the river? a. yes b. No
7. Do you defecate around the river? a. yes b. No
8. Do you immediately dry your body after swimming? a. yes b. No
9. Do you fetch river water for cooking/drinking? a. yes b. No
10. Do you participate in mass drug treatment for intestinal parasitosis?

a. yes b. No
