# Supplementary material for: Prevalence of intestinal parasites versus knowledge, attitude and practices (KAPs) with special emphasis to Schistosoma mansoni among individuals who have river water contact in Addiremets town, Western Tigray, Ethiopia
Source: PLoS One. 2018 Sep 25;13(9):e0204259. doi: 10.1371/journal.pone.0204259 (PMC6155513; doi:10.1371/journal.pone.0204259)
Supplement: S2 File — (PDF) [file pone.0204259.s002.pdf]

Questioner (Amharic version)

ኣዲስ ኣበባ ዩኒቨርስቲ የጤና ሳይንስ ኮሌጅ፤ ኣላይድ ጤና ሳይንስ ትምህርት ቤት የህክምና ላቦራቶሪ ሳይንስ ትምህርት ክፍል

የጥናቱ ኣላማ: በኣዲረመፅ ከተማ ውስጥ ከወንዝ ጋር ግንኙነት ካላቸው ሰዎች ስንቶቻቸው ናቸው አንጀት የሚያጠቃ ብልሃርዝያ እና ሌሎች አንጀት የሚያጠቁ ትላትል ያላቸው ፤ ሰዎቹ ስለ ብልሃርዝያ ያላቸው እውቀት፣ እምነት እና ተግባር ለማወቅ ነው።

ክፍል አንድ: ማህበራዊ እና ግለ-ታሪክ

የመጠይቁ ቁጥር____________

I. ፆታ: 1. ወንድ 2. ሴት እድሜ ___________________

II. ሀይማኖት፡ 1. ኦርቶዶክስ 2. ሙስሊም 3. ፕሮቴስታንት 4. ሌላ ካለ ይግለፁ___________

III. ስራ ፡ 1. ቅጥረኛ 2. ያልተቀጠረ (ስራ የልለው) 3. ተማሪ 4. ገበሬ 5. የቤት እመቤት 6. ሌላ ካለ ይግለፁ ________

IV. የትምህርት ሁኔታ ፡ 1. ያልተማረ 2. ኣንደኛ ደረጃ (1-8) 3. ሁለተኛ ደረጃ (9-10) 4. ፕሪፓራቶሪ (11-12) 5. ዩኒቨርስቲ 6. ማንበብ እና መፃፍ ብቻ

V. በኣዲረመፅ ከተማ ውስጥ የቆዩበት ጊዜ ______________________________

VI. ወደ ኣዲረመፅ ከተማ ከመምጣትዎ በፊት የነበሩበት ቦታ _____________________________

ክፍል ሁለት: ከወንዝ ውሃ ጋር ያላቸው ንኪኪ

1. መፀዳጃ ቤት ኣልዎት? ሀ. አዎ ለ. የለኝም

2. ለጥያቄ ቁጥር 1. መልስዎ አዎ ከሆነ ሁሉም የቤተሰብዎ አባል መፀዳጃ ቤት ይጠቀማል?

ሀ. አዎ ለ. አይጠቀሙም

3. ለጥያቄ ቁጥር 1. መልስዎ የለኝም ከሆነ የት ነው የሚፀዳዱት?

ሀ. ወንዙ ዳር ለ. ከወንዙ ራቅ ያለ ቦታ

ሐ. በፖፖ ተጠቅሜ ወደ ወንዙ እጥለዋለሁ

4. ለምግብ ማብሰያ እና ለመጠጥ የሚሆን ውሃ ከማይፃእዳ ወንዝ ይቀዳሉ?

ሀ. ኣዎ ለ. አልቀዳም

5. በቀን ስንት ጊዜ ይቀዳሉ? ....

6. ማይፃእዳ ወንዝ ውስጥ ይዋኛሉ? ሀ. አዎ ለ. አልዋኝም

7. በሳምንት ለምን ያህል ጊዜ ይዋኛሉ?

ሀ. በሳምንት ሶስት ጊዜ ለ. በሳምንት ሁለት ጊዜ ሐ. በሳምንት አንድ ጊዜ

8. በወንዙ ውስጥ ለምን ያህል ሰዓት ይዋኛሉ? .... ሰዓት

9. ስንት ሰኣት ለይ ነው የሚዋኙት?

ሀ. 1፡30-3፡30 ለ. 3፡30-5፡30 ሐ. 5፡30-7፡30 መ. 7፡30-9፡30 ሰ. 9፡30-11፡30

10. ከዋኙ በኋላ ገላዎ ወድያውኑ በፎጣ ያደርቃሉ?

ሀ. አዎ ለ. አላደርቅም

11. በማይፃእዳ ወንዝ ሲያልፉ ከወንዙ ጋር ይነካካሉ? ሀ. አዎ ለ. አልነካካም

12. ልብስዎ ማይፃእዳ ወንዝ ውስጥ ያጥባሉ? ሀ. አዎ ለ. አላጥብም

13. ገላዎ የት ነዉ የሚታጠቡት? ሀ. ማይፃእዳ ወንዝ ለ. ቤት ሐ. የህዝብ ሻወር

14. ለጥያቄ ቁጥር 13. መልስዎ ቤት ውስጥ ከሆነ ውሃው ከየት ነው የሚያገኙት?

ሀ. ማይፃእዳ ወንዝ ለ. ከቧንቧ ውሃ

ሐ. የተከለለ የምንጭ ውሃ መ. የተከለለ ከጉድጓድ ውሃ

15. ጫማ ያደርጋሉ? ሀ. አዎ ለ. አላደርግም

16. ለጥያቄ ቁጥር 15. መልስዎ አዎ ከሆነ ምን ዓይነት ጫማ ነው የሚያደርጉት?

ሀ. ነጠላ ጫማ ለ. ሸፍን ጫማ ሐ. ቡትስ

ክፍል ሦስት: እውቀት፤ ኣመለካከት፤ ልምድ

ሀ. የጥናቱ ተሳታፊዎች ስለ ብልሀርዝያ እና ሌሎች ኣንጀት የሚጠቁ ትላትሎች በሽታ ያላቸውን እውቀት ለመገምገም የተዘጋጀ መጠይቅ

1. አንጀትን ስለ የሚያጠቁ ትላትሎች ሰምተው ያውቃሉ? ሀ. አዎ ለ. አላቅም
2. አንጀትን ስለ የሚያጠቁ ትላትሎች እንዴት ነው ያወቁት?

ሀ. ሬድዮ ለ. ከጤና ተቋም

ሐ. ከጓደኛ መ. ሌላ ካለ ይግለፁ

3. ለጥያቄ ቁጥር 1 መልስዎ አዎ ከሆነ, የተወሰኑትን አንጀትን የሚያጠቁ ትላትሎች መጠቀስ የችላሉ?

____

4. አንጀትን ስለ የሚያጠቃ ብልሃርዝያ ሰምተው ያውቃሉ? ሀ. አዎ ለ. አላቅም

5. ብልሃርዝያ እንዴት ነው የሚተላለፈው?

ሀ. በተበከለ ወንዝ ውሃ ውስጥ በመዋኘት ለ. የተበከለ ውሃ በመጠጣት

ሐ. በተበከለ ወንዝ ውስጥ በመጫወት መ. በቀንድ ኣውጣ

ሰ. በትንኝ ረ. በተበከለ ምግብ

6. ይሄ ጥያቄ ለጥያቄ ቁጥር 5. መልስዎ ‘መ’ ከሆነ ቀንድ ኣውጣ የት ነው የሚኖረው?

ሀ. ወንዝ ውስጥ ለ. አፈር ውስጥ ሐ. አላውቅም

7. አንጀት የሚጠቃ የብልሃርዝያ በሽታ ዋና ዋና ምልክቶቹ ምንድን ናቸው? (ከአንድ በላይ መልስ መመለስ ይቻላል)

ሀ. ትኩሳት ለ. ራስ ምታት

ሐ. ድካም መ. ደረቅ ሳል

ሰ. የሆድ ህመም ረ. ተቅማጥ

ሸ. በሰገራ ውስጥ ደም መኖር ቀ. ኣላውቅም በ. ሌላ ካለ ይግለፁ

8. የብልሃርዝያ በሽታ ሊታከም ይችላል?

ሀ. አዎ ለ. አይታከምም

9. የብልሃርዝያ በሽታ መድሀኒት ከየት ቢያገኙ ነው የሚመርጡት?

ሀ. ከባህላዊ ህክምና ለ. ከጤና ተቋም

ሐ. ከመድሀኒት መደብር መ. ሌላ ካለ ይግለፁ

10. የብልሃርዝያ በሽታ መከላከል ይቻላል? ሀ. አዎ ለ. አይቻልም

11. ለጥያቄ ቁጥር 10. መልስዎ አዎ ከሆነ የብልሃርዝያ በሽታ እንዴት ነው የሚከላከሉት?

ሀ. መድሀኒት በመውሰድ ለ. በተበከለ የወንዝ ውሀ ውስጥ አለመታጠብ/አለመዋኘት

ሐ. መፀዳጃ ቤት መጠቀም መ. ንፁ የቧንቧ ውሃ በመጠቀም

ሰ. ወንዝ ዳር አለመፀዳዳት ረ. ሌላ ካለ ይግለፁ ሠ. አላውቅም

ለ. የጥናቱ ተሳታፊዎች ስለ ብልሀርዝያ እና ሌሎች ኣንጀት የሚጠቁ ትላትሎች በሽታ ያላቸውን አመለካከት ለመገምገም የተዘጋጀ መጠይቅ

1. አንጀትን የሚያጠቁ ትላትሎች በሽታ አደገኛ በሽታ ነው ብለው ያምናሉ? ሀ. አዎ ለ. አላምንም

2. አንጀትን ለሚያጠቁ ትላትሎች በሽታ መድሀኒት መውሰድ አስፈላጊ ነው ብለው ያምናሉ?

   ሀ. አዎ ለ. አላምንም

3. ሆድዎ ስያምዎት ወደ ጤና ተቋም መሄድ አስፈላጊ ነው ብለው ያምናሉ?

   ሀ. አዎ ለ. አላምንም

4. አንጀትን ለሚያጠቁ ትላትሎች በሽታ የባህል መድሀኒት መውሰድ ጥሩ ነው ብለው ያምናሉ?

   ሀ. አዎ ለ. አላምንም

5. አፈር ላይ መጫወት አንጀትን የሚያጠቁ ትላትሎች በሽታ ያስከትላል ብለው ያምናሉ?

   ሀ. አዎ ለ. አላምንም

6. ያልበሰሉ አትክልቶች መብላት አንጀትን የሚያጠቁ ትላትሎች በሽታ ያስከትላል ብለው ያምናሉ?

   ሀ. አዎ ለ. አላምንም

7. የብልሃርዝያ በሽታ አደገኛ በሽታ ነው ብለው ያምናሉ? ሀ. አዎ ለ. አላምንም

8. ለብልሃርዝያ በሽታ መድሀኒት መውሰድ አስፈላጊ ነው ብለው ያምናሉ?

   ሀ. አዎ ለ. አላምንም

9. በወንዝ ውስጥ መዋኘት/መታጠብ የብልሃርዝያ በሽታ ያስከትላል ብለው ያምናሉ?

10. የብልሃርዝያ በሽታ የምድን በሽታ ነው ብለው ያስባሉ? ሀ. አዎ ለ. አላስብም

ሐ. የጥናቱ ተሳታፊዎች ስለ ብልሀርዝያ እና ሌሎች ኣንጀት የሚጠቁ ትላትሎች በሽታ ያላቸውን ልምድ ለመገምገም የተዘጋጀ መጠይቅ

1. ጥሬ ሰጋ/ አትክልቶች ይበላሉ? ሀ. አዎ ለ. አልበላም

2. ምግብ ከመብላትዎ በፊት እጅዎትን ይታጠባሉ? ሀ. አዎ ለ. አልታጠብም

3. የሆድ ህመም ሲሰማዎት ወደ ጤና ተቋም ይሄዳሉ? ሀ. አዎ ለ. አልሄድም

4. አንጀትን ለሚያጠቁ ትላትሎች በሽታ መድሀኒት ይወስዳሉ?

ሀ. አዎ ለ. አልወስድም

5. ልብስዎ በወንዙ ውስጥ ያጥባሉ? ሀ.አዎ ለ. አላጥብም

6. በወንዙ ውስጥ ይታጠባሉ/ይዋኛሉ? ሀ. አዎ ለ. አልታጠብም/አልዋኝም

7. በወንዙ አካባቢ ይፀዳዳሉ? ሀ. አዎ ለ. አልፀዳዳም

8. ከዋኙ በኋላ ወድያውኑ ገላዎን ያደርቃሉ? ሀ. አዎ ለ. አላደርቅም

9. ለምግብ ማብሰያ/ለመጠጥ ውሃ ከወንዝ ይቀዳሉ? ሀ. አዎ ለ. አልቀዳም

10. አንጀትን ለሚያጠቁ ትላትሎች በሽታ መድሀኒት በህብረት በሚሰጥበት ጊዜ ተሳትፈው ያውቃሉ? ሀ. አዎ ለ. አላቅም
